# Occupational Burnout Is Linked with Inefficient Executive Functioning, Elevated Average Heart Rate, and Decreased Physical Activity in Daily Life - Initial Evidence from Teaching Professionals

**DOI:** 10.3390/brainsci12121723

**Published:** 2022-12-16

**Authors:** Mia Pihlaja, Pipsa P. A. Tuominen, Jari Peräkylä, Kaisa M. Hartikainen

**Affiliations:** 1 Behavioral Neurology Research Unit, Tampere University Hospital, 33521 Tampere, Finland; 2 Faculty of Medicine and Health Technology, Tampere University, 33520 Tampere, Finland; 3 Social Services and Health Care, Tampere University of Applied Sciences, 33520 Tampere, Finland

**Keywords:** biomarkers, brain health, burnout, depression, executive functions, heart rate, heart rate variability, physical activity, physiology, wearable devices

## Abstract

Burnout is becoming a global pandemic jeopardizing brain health, with a huge impact on quality of life, available workforce, and the economy. Knowledge of the impact of burnout on cognition, physiology, and physical activity (PA) in daily life allows for an improved understanding of the health consequences and everyday ramifications of burnout. Twenty-eight volunteers participated in a three-day recording of daily physiology and PA, including heart rate (HR) and daily steps, with a wearable device. They filled in questionnaires screening for burnout (BBI-15), depression (BDI), and executive functions (EFs) in daily life (BRIEF-A). The subjects with burnout had more challenges in EFs, higher average HRs and lower numbers of steps in daily life than those without it. The BBI-15 scores correlated positively with the BDI scores and BRIEF-A indices and negatively with the awake HR variability (HRV) and daily steps. The metacognition index correlated negatively with the HRV. In conclusion, burnout is linked with compromised EFs along with alterations in cardiac physiology and PA in daily life. Such alterations may be easily detected with wearable devices, opening possibilities for novel biomarkers of burnout and other neuropsychiatric disorders. We suggest that physical activity and heart and brain health are intimately intertwined and that burnout interacts with each of them bidirectionally.

## 1. Introduction

Occupational burnout is a stress-related condition associated with many physical and mental health challenges and risks [1]. While burnout has a measurable impact on physiology, including cardiac and brain functions, reliable physiological biomarkers of burnout are currently lacking. The diagnoses of burnout rely mainly on questionnaires assessing psychological constructs such as exhaustion, cynicism toward work, and lack of professional self-esteem [2]. Diagnosis based on self-evaluation questionnaires lacks assessment of alterations of physiological or cognitive functions due to burnout [3]. Wearable devices allowing for continuous measurement of physical activity (PA) and cardiovascular physiology in daily life could provide a basis for novel biomarkers of burnout, contributing to improved diagnostics and targeted interventions. A better understanding of the impact of burnout on cognitive and physiological functions is important not only for improved diagnostics but also for improved insight into the interaction of burnout and daily life.

In occupational burnout, the stress reaction is prolonged without adequate recovery. Stress reaction is beneficial when facing a challenging event or a task, as it allows the body to mobilize both mental and physical resources. As a critical part of the stress reaction, the sympathetic nervous system is activated, resulting in changes in cardiovascular physiology including an increase in heart rate (HR). During the following recovery phase, the parasympathetic nervous system is activated, counteracting many of the effects of the sympathetic activation and helping regain homeostasis. Lack of an adequate recovery phase may lead to dysregulation of the autonomic nervous system with sustained sympathetic activation and reduced parasympathetic activity, thought to be one of the main mechanisms behind the untoward health consequences of prolonged stress and burnout.

This disbalance or a balance between sympathetic and parasympathetic activity is reflected in the heart rate variability (HRV). Continuous HR and HRV can be measured and analyzed with different wearable devices. In previous studies, subjective stress has been found to be associated with device-measured stress, with a lower HRV indicating higher stress and higher HRV indicating recovery and lower stress [4]. Furthermore, previous studies have found elevated resting HR levels [5] and lower HRV [6,7] in subjects with burnout compared with healthy controls. To that end, HRV, measuring the balance of the autonomic nervous system (ANS) (i.e., sympathetic and parasympathetic activation) has been suggested as a potential biomarker of burnout.

While short-term stress usually enhances performance and helps to adapt to changing and possibly threatening conditions, prolonged stress has many negative health consequences. Longitudinal studies have shown that burnout is associated with insomnia [8,9] and depressive symptoms [10,11,12]. In addition, burnout has been identified as a predictor of psychotropic and antidepressant treatments [13,14] and hospital admissions due to mental disorders [15,16]. Longitudinal studies have further shown that burnout is an independent risk factor for several diseases [1]. Associations with hypercholesterolemia [17] and type 2 diabetes [18] have been found. Hospitalizations due to cardiovascular diseases have been linked to burnout [15]. In addition, a greater incidence of coronary heart disease in subjects with burnout has been reported [19]. Many symptoms, such as musculoskeletal pain [20], gastrointestinal and respiratory symptoms and conditions [21], severe injuries [22], and prolonged fatigue [23] have been linked with burnout. Even mortality below the age of 45 years has been associated with burnout [24]. Considering the vast variety of potentially severe health consequences in addition to the detrimental impact on quality of life, it is important to detect burnout promptly and better understand how burnout impacts daily life.

Several previous studies have studied the balance between PA, stress, and recovery [4,25,26,27,28,29]. A higher level of PA has been associated with a reduction in perceived stress in cross-sectional [4] and longitudinal studies [27,29]. Previous studies have shown that a low volume of exercise is linked with increased risk of burnout [25,26]. The relationship between PA and burnout is likely multifaceted. A low volume of PA may be both a contributing factor and a consequence of burnout. For example, fatigue or compromised executive functions (EFs) in burnout may result in sedentary life choices linked with immediate reward over future benefit.

To make healthy life choices which support recovery and adequate PA, one needs to have efficiently functioning EFs that allow for delaying gratification. EFs are higher cognitive functions that control cognition, emotions, and behavior [30,31]. The efficiency of these functions may vary even in healthy subjects from day to day, depending on several factors such as vigilance, motivation, and brain strain. Wiehler et al. [32] reported that engaging in tasks demanding cognitive control increased the level of glutamate in the lateral prefrontal cortex and resulted in nonoptimal decision making biased by short-term rewards. The study showed a link between cognitive fatigue, alteration in glutamate levels, and reduced cognitive control favoring low-effort actions and short-term rewards. In other words, cognitive strain leads to alterations in neurotransmission and compromises performance in tasks requiring EFs. Postponing a reward relies heavily on well-functioning EFs.

Impaired EFs are a frequent concomitant of many neuropsychiatric disorders such as attention deficit disorder (ADHD) and depression and have also been linked with burnout. Previous studies have shown that burnout is associated with deficits in memory functions, attention, and executive function. On the other hand, cognitive deficits and specifically executive dysfunction could lead to burnout when the cognitive demands at work exceed a worker’s cognitive capacity [33]. To that end, impaired EFs may predispose one to burnout, and burnout may further impair EFs, creating a vicious cycle.

EFs are also important for work ability in most modern occupations. Impaired ability to work due to occupational burnout is most likely not only linked to psychological symptoms of burnout, such as low professional self-esteem, exhaustion, and cynicism toward work, but may also be due to impaired efficiency of EFs. In addition to impacting work ability and efficiency at work, EFs contribute to healthy life choices, appropriate emotional control, euthymic mood, flexible behaviors, and successful daily living in general. To that end, it is critical to better understand the impact of burnout on EFs in daily life.

The aim of the current study is to shed light on the interaction of burnout with daily life, more specifically with EFs, HR, HRV, and PA in daily life. We expected burnout to be linked with impaired EFs in daily life. Furthermore, we expected burnout to be associated with increased HR and reduced HRV, reflecting increased sympathetic activity. In addition, as in some earlier studies [25,26], a low volume of exercise in everyday life has been linked with greater risk of burnout, and we expected smaller numbers of daily steps in subjects with burnout in contrast to those without it. Finally, we aim to illuminate possible links between burnout, depression, EFs, HR, HRV, and PA.

## 2. Materials and Methods

The participants were teaching professionals, namely teachers and principals, working in the city of Tampere in Finland, a target group of the Sustainable Brain Health project led by Tampere University of Applied Sciences and funded by the Finnish Ministry of Social Affairs and Health from the European Social Fund’s Programme for Sustainable Growth and Jobs 2014–2020 Finnish structural fund. The study was reviewed and approved by the Ethical Committee of the Pirkanmaa Hospital District (approval number: R20094), and participants provided their written informed consent according to the Declaration of Helsinki governing the use of human subjects.

A total of 54 neurologically healthy subjects were recruited into the study by email newsletter. All volunteers received written information about the study, and they had the opportunity to make inquiries about the study from researchers before signing the consent form. Exclusion criteria were history of brain injury, neurological, psychiatric, or cardiac disease, or medications impacting HR or cognition.

The participants completed three inventories screening for burnout (Bergen Burnout Indicator 15 (BBI-15)) and depression (Beck’s Depression Inventory (BDI)), and measuring EFs in daily life (Behavioral Rating Inventory of EFs—Adult Version (BRIEF-A)). Computer-based experimental and integrated testing of EFs based on a Go/No-go reaction time test (Executive reaction time (RT) test) [34,35] and simultaneous EEG recordings were conducted at the Behavioral Neurology Research Unit of Tampere University Hospital. Subjects received feedback on the results of the inventories, and they were directed to evaluation by their occupational health nurse or doctor if any health concerns were detected.

Subjects were assigned into burnout and non-burnout groups based on the BBI-15 scores. All subjects with a score suggesting at least mild burnout (percentile > 75) were assigned to the burnout group. Thus, in the burnout group, there were subjects who had BBI-15 score indicating anything from mild to severe burnout, and in the non-burnout control group, there were only the subjects without any indication of burnout in their BBI-15 scores (percentile < 75).

**Figure 1:**
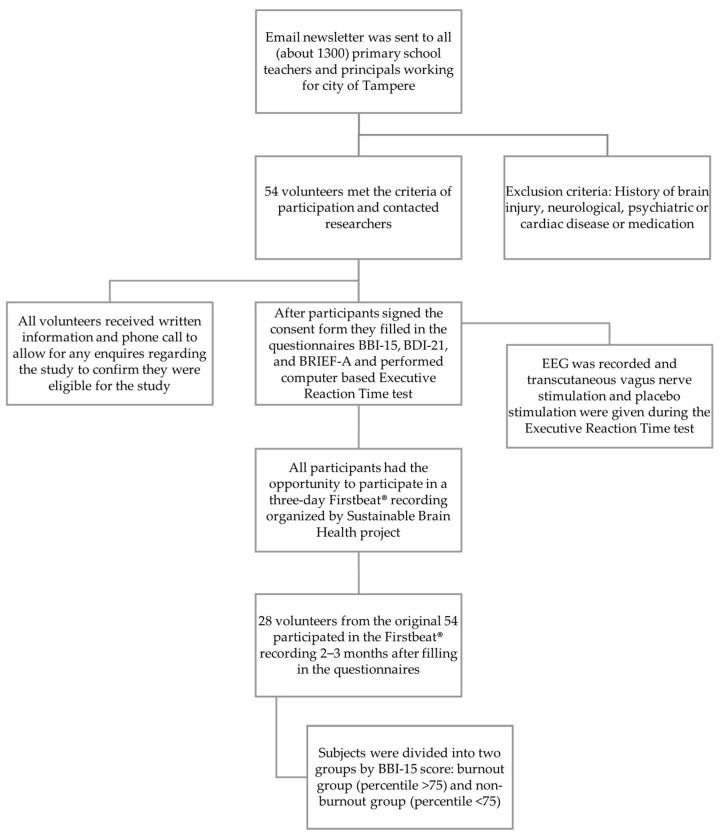
Recruitment and research process flowchart.

All subjects had an opportunity to participate in a three-day Firstbeat^®^ recording (Firstbeat Technologies Ltd., Jyväskylä, Finland) [36], including two working days and one day off of work. Twenty-eight subjects from the original group of 54 subjects participated. Firstbeat^®^ recordings were performed 2–3 months after filling in the inventories and participating in the EEG recording. In this study, we present the results obtained from the inventories and the Firstbeat^®^ measurements from these 28 subjects. The recruitment and research processes are illustrated in the flowchart (Figure 1).

### 2.1. Bergen Burnout Indicator 15 (BBI-15)

The Bergen Burnout Indicator 15 (BBI-15) consists of 15 statements related to 3 main symptoms of burnout: cynicism, lack of professional self-esteem, and exhaustion. The subjects respond on a scale from 1 to 6 reflecting how much they agree with the statements. The response scores are summed up for each subdivision separately. Furthermore, subdivision scores are summed up to find an overall score for burnout. Each score is compared with age- and gender-normalized values to obtain the overall severity of burnout, as well as the level of each dimension of burnout. The level of burnout is obtained from a table, where the scores are divided into percentiles: no burnout = 25–70, mild = 75–80, moderate = 85–90, and severe = 95–97.5. The BBI-15 is widely used in the Finnish occupational healthcare system, and there is a strong correlation between the BBI-15 and the Maslach Burnout Inventory scores [37].

### 2.2. Beck’s Depression Inventory 21 (BDI)

Beck’s Depression Inventory 21 (BDI) consists of 21 multiple-choice statements on symptoms of depression. Responses are rated from zero to three and summed up to find the overall score. The score refers to the level of depression symptoms, with scores of 0–12 indicating no depression, 13–18 indicating mild depression, 19–29 indicating moderate depression, and over 30 indicating severe depression [38].

### 2.3. Behavioral Rating Inventory of Executive Functions—Adult Version (BRIEF-A)

BRIEF-A is a validated self-assessment tool consisting of 75 statements on executive functioning in daily life. Responses are given based on how frequently the challenge related to a specific executive function in the statement is causing problems in daily living: never, sometimes, or frequently. Responses are summed up to obtain the raw scores for nine different clinical EF scales, two indices (metacognition (MI) and behavior regulation (BRI) indices), and a global executive composite (GEC) derived by combining all these scales. The MI consists of initiation, working memory, planning or organization, task monitoring, and organization of material scales, and the BRI consists of emotional control, shifting, inhibition, and self-monitoring scales. The GEC is the aggregate index of all nine scales. The raw scores are transformed into *T*-scores, which are age-normalized values with a mean of 50 and standard deviation of 10 in a normative sample. For example, a *T*-score of 70 is two standard deviations above the normative sample mean and equals the scores of approximately 95% of responders in a normative sample. *T*-scores were used in the analyses. *T*-scores over 65 are considered abnormal (i.e., they differ by more than 1.5 standard deviations from the normative sample mean) [39].

### 2.4. Firstbeat^®^ Well-Being Survey

The Firstbeat^®^ Bodyguard 2-device (Firstbeat Technologies Ltd., Jyväskylä, Finland) was used to measure HR, HRV, and tri-dimensional acceleration signals between August and September 2021. There were two electrodes attached to participant’s chest at two locations. The device was used continuously for three days and nights, except during water sports and while showering. The recording time (session total time) was indicated in minutes. During the recording period, the device recorded the HR in beats per minute and HRV as the changes between R-R-intervals using the root mean square of the standard deviation (RMSSD) in milliseconds [40]. The average HR is the average over the entire recording, MaxHR is the highest 10 s average HR, and MinHR is the lowest 10 s average HR. Beat-by-Beat RMSSD is the average variation in consecutive R-R-intervals, and it was detected during waking hours (HRV-Awake) and during sleep (HRV-Sleep), separately. Sleeping time was based on a diary, and if not documented, the device-detected sleeping time in minutes was used. Furthermore, the built-in accelerometer collected the tri-axial acceleration signal with a 200 Hz sampling frequency and 8 g (Earth’s gravity) measurement range caused by any movements. Steps were detected using an acceleration signal. The Firstbeat^®^ analysis program processed the collected raw data, providing analyzable data for stress, recovery, sleep, sedentary behavior, PA, and steps.

### 2.5. Statistical Analyses

Group differences were analyzed using the two-tailed *t*-test and equality of the variances confirmed with the F-test. Correlation analysis was conducted with partial Spearman’s rank correlation, controlling for gender and the BBI-15 score, BDI score, or both. Here, *p*-values < 0.05 were considered statistically significant. F- and *t*-tests were performed using Microsoft^®^ Excel^®^ for Microsoft 365 MSO (version 2209, build 16.0.15629.20152) and its tools for the mean, standard deviation, and two-tailed *t*-test. Correlation analysis was performed with R (version 4.2.0 (22 April 2022 ucrt) [41] and the ppcor-package [42].

## 3. Results

### 3.1. Groups and BBI-15

Based on the BBI-15 inventory scores, 12 women and 2 men were assigned into the burnout group, and 14 women were assigned to the non-burnout group. The mean age of the non-burnout group was 42.1 (SD = 10.6) years, and that of the burnout group was 43.9 (SD= 8.8) years. The groups did not differ from each other in age (*p* = 0.63), years of education (non-burnout = 18.7 years (SD = 2.6), burnout = 18.9 years (SD = 1.15, *p* = 0.88)), or Firstbeat^®^ recording time (non-burnout = 4250.4 (SD = 118.8) minutes, burnout = 4209.4 (SD = 289.1) minutes, *p* = 0.63).

In the burnout group, five subjects had severe burnout, six had moderate burnout, and three had mild burnout according to BBI-15 scores. The mean BBI-15 score was 32.21 (SD = 7.50) in the non-burnout group and 57.50 (SD = 6.87) in the burnout group. The exhaustion score in non-burnout group was 13.36 (SD = 4.29), and in burnout group, it was 21.43 (SD = 4.31). The cynicism score in the non-burnout group was 7.79 (SD = 2.61), and in the burnout group, it was 15.93 (SD = 3.79). Finally, the score for the decline in professional self-esteem in the non-burnout group was 11.07 (SD = 3.05), and in the burnout group, it was 20.14 (SD = 3.37).

### 3.2. BDI

The BDI scores differed significantly between the groups, with the mean score reflecting no depression in the non-burnout group and mild depression in the burnout group (non-burnout = 3.64 (3.62), burnout = 15.6 (6.39) $t_{26}$ = −6.12, *p* < 0.001).

### 3.3. BRIEF-A

The non-burnout and burnout groups differed significantly in terms of the Metacognition Index and Behavior Regulation Index scores, as well as in the Global Executive Composite scores. Moreover, the differences in all nine individual clinical scales were significant. The BRIEF-A means, standard deviations, and *t*-test results are listed in Table 1.

### 3.4. Firstbeat^®^ Recording

The Firstbeat^®^ analysis showed significant differences between the non-burnout and burnout groups in terms of average HR and number of steps, with the burnout group having a higher HR and lower number of daily steps. There was a tendency toward lower HRV-Awake values in the burnout group. There were no statistical differences between the groups in the lowest daily HR (MinHR), the highest daily HR (MaxHR), or the total amount of sleep. The Firstbeat^®^ recordings’ means, standard deviations, and *t*-test results are listed in Table 2.

### 3.5. Spearman´s Partial Rank Correlation for Burnout, BRIEF-A Indices, and Firstbeat^®^ Results

The BBI−15 score correlated significantly with the BDI (ρ = 0.83, *p* < 0.001), BRIEF-A composite indices (BRI: ρ = 0.61, *p* < 0.001; MI ρ = 0.75, *p* < 0.001, and GEC ρ = 0.73, *p* < 0.001), minimum HR (ρ = 0.38, *p* = 0.052), HRV-Awake (ρ = −0.44, *p* = 0.021), and number of daily steps (ρ = −0.44, *p* = 0.020) with gender controlled for (Table 3).

HRV-Sleep and HRV-Awake correlated negatively and significantly with the GEC and MI, and correlation with the BRI approached significance. There was also a negative correlation between the daily steps and MI (ρ = −0.42, *p* = 0.031) and a negative correlation approaching significance between the daily steps and GEC (ρ = −0.37, *p* = 0.059). Negative correlation between the amount of sleep and MI was approaching significance (ρ = −0.37, *p* = 0.058).

As the BBI-15 and BDI scores were strongly correlated, probably reflecting partly overlapping symptoms of depression and burnout, we conducted further post hoc correlation analysis with the BBI-15 score (Table 4), BDI score (Table 5), or both (Table 6) being controlled for. In all correlation analyses, gender was also controlled for.

When the BDI score and gender were controlled for, the BBI-15 score correlated positively with the MI (ρ = 0.47, *p* = 0.016) and negatively with the daily steps (ρ = −0.40, *p* = 0.042). On the other hand, when the BBI-15 score and gender were controlled for, the BDI correlated positively with the BRI (ρ = 0.39, *p* = 0.047).

When the relationship between the BRIEF indices and Firstbeat^®^ variables was analyzed while controlling for gender, the BDI and BBI-15 scores and HRV-Sleep correlated negatively with the MI (ρ = −0.42, *p* = 0.037).

## 4. Discussion

The aim of the current study was to improve understanding of burnout on EFs, physiology reflecting stress and recovery, and the level of PA in everyday life. We used physiological markers easily measurable by variety of wearable devices such as HR, HRV, and the number of steps and assessed whether they differed between subjects with and without burnout. We further investigated whether these measures correlated with the burnout symptom scores or with executive functions in daily life. We found out that burnout was linked with subjective challenges in daily tasks requiring EFs. Furthermore, subjects experiencing burnout symptoms had lower levels of PA, higher average HRs, and a trend toward a lower HRV during wakefulness compared with the subjects without burnout symptoms. In addition, correlations between the BBI-15 score, BRIEF indices, HRV, and number of daily steps were found, suggesting a tight link between burnout, higher cognitive control functions, the activity balance of the ANS, and the level of PA in daily life.

The subjects with burnout experienced more subjective challenges in executive functioning in everyday life compared with those without it. The challenges were, however, subtle, and the mean *T*-scores did not reflect clinical executive dysfunction. Challenges in different cognitive functions due to burnout have been reported in many earlier studies, especially in EFs, attention, and memory [33,43]. However, findings are inconsistent. Van Der Linden et al. [44] suggested that burnout might be associated with deficits in sustained attention and inhibition, while Jonsdottir et al. [45] showed possible deficits in executive control tasks, attention span, working memory, learning, and episodic memory in a burnout group compared with healthy controls. Further, a longitudinal study by Feuerhahn et al. [46] suggested that impaired cognitive performance among teachers with burnout might be caused by deficits in EFs. On the other hand, neither Österberg et al. [47] nor McInerney et al. [48] found any significant association between neurocognitive performance and burnout. However, in a 1.5 year follow-up of former burnout patients, Österberg et al. [49] found that there might be a slight but reversible cognitive impairment in burnout.

According to Gavelin et al., cognitive impairments in burnout might be prefrontal in nature i.e., executive dysfunction could cause impairments in other cognitive domains by impairment in cognitive control [43]. Furthermore, structural and functional changes in the prefrontal cortex, as well as in the amygdala and the striatum, have been found in many neuroimaging studies [50,51,52], providing the underlying neural basis for executive dysfunction related to burnout. Structural changes in brain regions might be caused by elevated glucocorticoid levels caused by the prolonged uncontrollable stress underlying burnout [53]. Interestingly, Beck et al. showed that executive functioning might recover to the level of the healthy controls after acute burnout when subjects with burnout perform regular aerobic exercise training for 12 weeks [54]. Changes in burnout severity and executive function performance were not related [54].

A difference in the daily amount of PA between subjects with and without burnout was seen in this study. There was a lower number of steps in the subjects with burnout in contrast to those without it in the three-day recording, and there was a negative correlation between the number of steps and burnout symptoms. It is possible that burnout and challenges in EFs decrease PA. When burnout was controlled for, significant correlations between the BRIEF-A indices and steps were not found. Alternatively, it could be that low PA increases the risk of burnout or increases perceived burnout symptoms. This is in line with previous studies which have found that increased PA decreases burnout symptoms effectively [54], and on the other hand, a small amount of PA increases the risk of burnout [25,26]. Furthermore, Toker and Biron (2012) reported that there was a greater increase in burnout and depression in follow-ups with subjects with low-intensity PA, while there was little to no increase in those engaging in high-intensity PA [12].

In addition to the positive effects of PA on burnout symptoms [12,54,55], previous studies have suggested that increased PA improves cognitive functioning [56,57,58]. Our results are in line with this, as a higher level of PA in daily life was linked with less challenges in EFs in burnout. Interestingly, there was also a trend toward more efficient EFs with a greater amount of sleep. To that end, a physically active lifestyle along with an adequate amount of sleep may protect EFs in subjects with burnout. Furthermore, when the BBI-15 and BDI scores and gender were controlled for, higher HRV during sleep and while awake was linked with less challenges in executive functions in daily tasks requiring metacognitive functions, including working memory.

Considering the positive effects of PA on burnout symptoms and PA and sleep on EFs, as well as the very limited treatment options for burnout and executive dysfunction on the other hand, assessing the level of PA and the amount of sleep in daily life with wearable devices could be beneficial when evaluating risk for burnout and when planning treatment or rehabilitation. The number of steps is a very easy and accessible tool for basic evaluation of PA in everyday life, and it is possible to set very concrete and individual goals during rehabilitation if needed. More research is needed to find out the optimal level of PA to improve burnout symptoms and EFs. Of course, one needs to also consider the possibility that a low number of steps is a consequence of burnout and the associated challenges in EFs.

Inefficient EFs may be the reason for choosing an immediate reward, such as a sedentary lifestyle over a future reward of health benefits, such as through physical activity. There was a significant difference in the average HR and a trend toward significance in the HRV during wakefulness (i.e., burnout was linked with increased average HR and decreased HRV during daytime hours). Burnout symptoms also correlated significantly with HRV-Awake, and the correlation with minimum HR and HRV-Sleep approached significance. However, when controlling for depression symptoms, these correlations were no longer detected. Instead, burnout symptoms correlated negatively with the number of steps and positively with the MI. In other words, the more burnout symptoms, the lower the level of PA, and the more challenges in daily tasks requiring EFs related to metacognition.

In line with chronic stress in burnout, sympathetic nervous system activity related to physiological changes was seen in the subjects with burnout. When the sympathetic nervous system is more active than the parasympathetic nervous system, the HR increases and HRV decreases, as seen in the burnout group in the current study. Similar findings have been reported in previous studies [5,6], and HRV has been widely acknowledged as an objective marker of stress [59]. Changes in HR and HRV in daily living detected with wearable devices could potentially be used in the evaluation of risk of burnout, burnout severity, and recovery from burnout. However, caution is warranted, and more research needs to be conducted to assess how HR and HRV can be used reliably in individual assessments, with many other confounding factors affecting these measurements.

There were intriguing results from the current study emphasizing the intimate interaction between the heart, ANS, and the brain. With burnout scores and gender controlled for, higher HRV was linked with less challenges in daily tasks requiring metacognition. Metacognition correlated negatively with HRV-Sleep and HRV-Awake, and negative correlation between EFs in general and HRV-Sleep approached significance. When controlling for both the BBI-15 and BDI scores and gender, there was still a significant positive correlation between HRV-Sleep and the MI. These results highlight how EFs reflect health and wellbeing in general and, on the other hand, how health and wellbeing are reflected in more efficiently functioning EFs. In a relaxed state and in the recovery phase, EFs and especially metacognition seem to be more efficient, while stressful or threat-related events may capture limited executive function resources, compromising performance in other tasks requiring EFs [60]. 

HRV reflects the balance of the ANS (i.e., the balance between the sympathetic and parasympathetic nervous systems). The ANS is controlled by the same neural circuits which are also crucial for cognitive and emotional processes [61,62]. Activating the vagus nerve, a critical component of the parasympathetic nervous system, with invasive or non-invasive methods has been linked with improved EFs. More specifically, improved working memory has been reported with invasive vagus nerve stimulation (iVNS) and potentially more efficient cognitive control with transcutaneous VNS (tVNS) [63,64]. Thayer and Lane et al. hypothesized that reduced cognitive flexibility would be caused by prefrontal hypoactivation and disinhibition of the amygdala, caused by sympathetic hyperactivation. In turn, amygdala activation would contribute to a decrease in HRV and an increase in HR [65]. On the other hand, parasympathetic activation leads to increased HRV and improved cognitive functions [65] in line with the current finding of higher HRV linked with better metacognitive functions. Our results are further in line with previous findings presented in the review by Forte et al., highlighting the influence of the ANS on cognitive functions [66]. In the future, HRV could possibly provide an additional data point in the assessment of EFs in burnout. In addition, the results of the current study point to a tight interconnection between higher brain functions and cardiac physiology, reflecting the activity balance of the ANS. Improving heart health improves brain health, and vice versa.

The strengths of this study include assessment of cognitive functions and continuous physiological measurements in daily life, rather than snapshot measures in the laboratory. Firstbeat^®^ assessment was carried out for three days, including two working days and one day off, and the mean values of these three days were used in the analyses. This gave better information on continuous physiology and the impact of burnout on it in real life compared with snapshot measurements. In addition, while objective measurements of cognitive performance with standardized neurocognitive tests are of great importance, it is equally important to assess the perceived challenges in EFs in daily life in the context of burnout. Some of the challenges may be subtle enough that they are not depicted with cognitive testing in structured environments. On the other hand, daily life tends to put far greater challenges on EFs and for extended periods of time, in contrast to short assessment with standardized tests of EFs. Standardized neurocognitive tests of EFs were originally designed for depicting substantial deficits observed in frontal brain injuries rather than subtle dysfunction of the fronto-subcortical circuits due to prolonged brain strain, stress, or burnout. To that end, it is of great value to assess perceived challenges in daily tasks relying on different EFs with a standardized questionnaire broadly used in the assessment of EFs in neuropsychiatric and other brain disorders. To our knowledge, this is the first study to assess perceived EFs in daily life along with objective measures of PA and cardiac physiology reflecting the activity balance of the ANS.

The weaknesses of this study relate to a cross-sectional design with a relatively small sample size and unbalanced groups in terms of gender. However, in the correlation analysis, gender was controlled for. Furthermore, the groups consisted only of teaching professionals. For these reasons, caution is warranted when generalizing results across genders and other professions. Furthermore, the burnout group was quite heterogenous, ranging from mild to severe burnout and possibly diluting some of the effects. In addition, there was a time gap between the physiological measurements and the assessment of perceived challenges in cognitive functions in daily life. The Firstbeat^®^ assessments were made 2–3 months after participants filled in the inventories. In theory, it is possible that there were alterations in burnout status in both the non-burnout as well as the burnout groups before Firstbeat^®^ assessment, potentially confounding the results.

Furthermore, there was the challenge of disentangling the impact of burnout from that of depression because of partly overlapping symptoms [54]. With the aim to disentangle the impact of burnout from depression, we conducted correlation analysis with the BBI-15 score, BDI score, or both controlled for. However, distinguishing the effects of burnout without depression confounding the results is not trivial, as some of the symptoms are shared by both disorders. Thus, controlling for depression may dilute some true effects of burnout. The burnout group had, on average, mild depression in the BDI screen for depression, which did not necessarily indicate they had mild clinical depression on average, but this may just reflect the fact that subjects with burnout score higher on depression screens due to partly overlapping symptom profiles in burnout and depression.

Another consideration relates to the indirect physiological measures used in the study that are thought to reflect the level of stress and the activity balance of the autonomic nervous system, namely HRV. No direct measures of stress response or sympathetic activation, such as cortisol levels or pupillary response, were measured. To that end, there could be other factors than stress and increased sympathetic activity contributing to the increase in HR and a trend toward decreased HRV in burnout (e.g., physical fitness level).

The BDI scores differed between groups and correlated with the BBI-15 scores, in line with Näätänen et al., who among others has previously shown a strong positive correlation between BBI-15 and BDI scores [37]. Yet, researchers suggest burnout and depression are distinct but overlapping and interacting neuropsychiatric conditions [67]. Our results provide further evidence for that with distinct EF profiles in burnout and depression, i.e. burnout linked with compromised metacognition and depression with challenges in emotional and behavioral regulation. While burnout and depression are frequent comorbidities, and burnout may be followed by depression, robust evidence for a causal relationship between depression and burnout is lacking. There is also a lack of longitudinal studies aiming to shed light on this relationship [68]. Depression is known to be associated with cognitive deficits and executive dysfunction [69,70]. On the other hand, efficient treatment of drug-resistant severe depression with electroconvulsive therapy is linked with alleviation of depression and with improved EFs [71].

The results from the current study also suggest depression and burnout are distinct neurocognitive disorders. We aimed to disentangle the effects of depression and burnout by conducting correlation analysis with the BBI-15 score, BDI score, or both controlled for. When gender and burnout were controlled for, the BDI score correlated with the BRI but not with the MI. In contrast, when gender and depression were controlled for, the BBI-15 score correlated with the MI but not with the BRI. The results from the current study point toward challenges in EFs in both depression and burnout but with the metacognitive abilities, including working memory, more compromised in burnout and behavioral and emotional regulation more compromised in depression. These different EFs rely on different fronto-subcortical networks, with the dorsolateral prefrontal cortex, mediodorsal nucleus of the thalamus, and their networks implicated in working memory [72] and the orbitofrontal cortex and its networks implicated in emotional and behavioral regulation [73,74,75]. To that end, distinct neural circuits may be involved in burnout and depression.

Considering the importance of EFs in several aspects of daily life [31], including stress regulation [25], the relationship between burnout and EFs has been sparsely investigated, and biomarkers for executive dysfunction [71] and burnout are sparse. Occupational burnout is becoming a global pandemic, jeopardizing brain health with a huge impact on quality of life, the available work force, and the economy. Even though it is linked with many similar symptoms and challenges such as depression and other neuropsychiatric disorders, including challenges in EFs, it has yet to be considered a neuropsychiatric disorder.

Modern working life demands efficient EFs to tackle emotional and cognitive strain and information load. More studies on the interaction of executive functioning, PA, and cardiac physiology in daily life are needed for burnout. Combining data from wearable devices reflecting the balance of the ANS and the level of stress with assessments of cognitive functions and perceived symptoms may allow for novel biomarkers of burnout. Such biomarkers would contribute to improved and timely diagnosis and allow for intervention studies aiming at efficient and targeted treatments. In addition, wearable devices and a combination of biomarkers obtained from such measurements will allow for novel insight into the impact of other neuropsychiatric disorders on stress and PA in daily life as well.

## 5. Conclusions

Burnout is linked with inefficient EFs in everyday life along with physiological alterations, reflecting an overactive sympathetic nervous system. Fronto-subcortical circuits provide the neural basis for EFs and are frequently compromised in neuropsychiatric disorders. We suggest that rather than merely being an occupational psychological condition, burnout should be considered a neurocognitive brain disorder, such as depression, with distinct fronto-subcortical neural circuits involved.

Higher HRV was linked with more efficient executive functions in daily life. These results underline the intimate interaction between heart and brain health. Furthermore, burnout was associated with a reduced number of steps, suggesting a low level of PA could be a risk factor or an indicator of burnout. In addition, PA could even be a potential treatment of burnout.

Combining measures reflecting cognitive function, experienced symptoms, and the activity balance of the ANS and PA in daily life could be a basis for novel biomarkers of burnout. Such biomarkers would contribute to improved and advanced diagnostics as well as allow for objective measures reflecting brain health and wellbeing in daily life for intervention studies on burnout and potentially in other neuropsychiatric disorders as well.

## Figures and Tables

**Table 1:** BRIEF-A mean scores for the composite score (GEC), the two indices (MI and BRI), and the nine individual clinical EF scales, with standard deviations and *t*-test results listed for the two groups (non-burnout and burnout) (*p* < 0.05 (*), *p* < 0.01 (**), and *p* < 0.001 (***)).

	Group	Mean (SD)	*t*	df	*p*
GEC	Non-burnout	43.71 (7.76)	−4.77	26	<0.001 ***
	Burnout	59.14 (9.29)			
MI	Non-burnout	45.36 (9.09)	−4.38	26	<0.001 ***
	Burnout	60.14 (8.77)			
Initiate	Non-burnout	46.29 (9.01)	−3.06	26	0.005 **
	Burnout	59.07 (12.75)			
Working memory	Non-burnout	48.43 (8.95)	−4.65	26	<0.001 ***
	Burnout	63.14 (7.74)			
Plan/ Organize	Non-burnout	43.5 (9.43)	−3.79	26	0.001 **
	Burnout	55.93 (7.85)			
Task monitor	Non-burnout	45.07 (7.95)	−4.12	26	<0.001 ***
	Burnout	58.71 (9.50)			
Organization of materials	Non-burnout	47.36 (9.56)	−2.39	26	0.025 *
	Burnout	56.71 (11.13)			
BRI	Non-burnout	42.5 (7.29)	−3.92	26	0.001 **
	Burnout	56.71 (11.43)			
Inhibit	Non-burnout	43.86 (5.70)	−2.96	18	0.008 **
	Burnout	54.86 (12.71)			
Shift	Non-burnout	42.86 (4.27)	−5.55	26	<0.001 ***
	Burnout	55.29 (7.15)			
Emotional control	Non-burnout	45.57 (8.25)	−2.97	26	0.006 **
	Burnout	57.71 (12.81)			
Self-monitoring	Non-burnout	41.43 (7.11)	−3.41	26	0.002 **
	Burnout	52.07 (9.27)			

**Table 2:** Results of Firstbeat^®^ recordings with group means, standard deviations, and *t*-test results (*p* < 0.05 (*)).

	Group	Mean (SD)	*t*	Df	*p*
MinHR (min^−1^)	Non-burnout	50.98 (3.86)	−1.91	26	0.068
	Burnout	54.10 (4.76)			
MaxHR (min^−1^)	Non-burnout	143.74 (11.77)	−0.19	26	0.860
	Burnout	144.62 (14.29)			
AvHR (min^−1^)	Non-burnout	73.12 (5.08)	−2.72	26	0.032 *
	Burnout	78.40 (7.07)			
HRV-Awake (ms)	Non-burnout	36.31 (14.48)	2.04	18	0.057
	Burnout	27.67 (6.51)			
HRV-Sleep (ms)	Non-burnout	47.86 (23.14)	1.72	17.8	0.100
	Burnout	36.24 (10.18)			
Steps	Non-burnout	7527.19 (2427.99)	2.22	26	0.035 *
	Burnout	5323.88 (2811.73)			
Sleep Total Time (min)	Non-burnout	480.40 (38.20)	1.02	19.5	0.320
	Burnout	457.60 (74.10)			

**Table 3:** Spearman´s partial rank correlation coefficients and statistical significances for BBI−15 score, BDI score, BRIEF-A indices, and Firstbeat^®^ results with gender controlled for (*p* < 0.05 (*), *p* < 0.01 (**), and *p* < 0.001 (***)).

*n* = 28	BBI−15	BDI	BRI	MI	GEC
BDI	0.83 (<0.001 ***)				
BRI	0.61 (<0.001 ***)	0.68 (<0.001 ***)			
MI	0.75 (<0.001 ***)	0.68 (<0.001 ***)	0.69 (<0.001 ***)		
GEC	0.73 (<0.001 ***)	0.72 (<0.001 ***)	0.88 (<0.001 ***)	0.94 (<0.001 ***)	
MinHR (min^−1^)	0.38 (0.052)	0.38 (0.052)	0.15 (0.447)	0.38 (0.053)	0.29 (0.144)
AvHR (min^−1^)	0.25 (0.208)	0.17 (0.385)	0.15 (0.463)	0.30 (0.123)	0.24 (0.225)
HRV-Sleep (ms)	−0.36 (0.065)	−0.41 (0.034 *)	−0.32 (0.106)	−0.54 (0.004 **)	−0.49 (0.009 **)
HRV-Awake (ms)	−0.44 (0.021 *)	−0.48 (0.011 *)	−0.36 (0.068)	−0.57 (0.002 **)	−0.52 (0.005 **)
Steps	−0.45 (0.020 *)	−0.28 (0.159)	−0.31 (0.120)	−0.42 (0.031 *)	−0.37 (0.059)
Sleep (min)	−0.26 (0.190)	−0.32 (0.108)	−0.15 (0.444)	−0.37 (0.058)	−0.28 (0.152)

**Table 4:** Spearman’s partial rank correlation coefficients and statistical significances for BBI-15 score, BRIEF-A indices, and Firstbeat^®^ results with gender and BDI score controlled for (*p* < 0.05 (*), and *p* < 0.001 (***)).

*n* = 28	BBI-15	BRI	MI	GEC
BRI	0.11 (0.589)			
MI	0.47 (0.016 *)	0.43 (0.027 *)		
GEC	0.34 (0.094)	0.77 (<0.001 ***)	0.88 (<0.001 ***)	
MinHR (min^−1^)	0.13 (0.542)	−0.15 (0.456)	0.18 (0.383)	0.03 (0.896)
AvHR (min^−1^)	0.19 (0.346)	0.04 (0.845)	0.26 (0.205)	0.17 (0.407)
HRV-Sleep (ms)	−0.04 (0.841)	−0.06 (0.774)	−0.39 (0.049 *)	−0.31 (0.120)
HRV-Awake (ms)	−0.09 (0.673)	−0.05 (0.822)	−0.38 (0.055)	−0.29 (0.153)
Steps	−0.40 (0.042 *)	−0.17 (0.417)	−0.32 (0.110)	−0.25 (0.215)
Sleep (min)	0.00 (0.98)	0.09 (0.670)	−0.22 (0.277)	−0.08 (0.681)

**Table 5:** Spearman´s partial rank correlation coefficients and statistical significances for BDI score, BRIEF-A indices, and Firstbeat^®^ results with gender and BBI-15 score controlled for (*p* < 0.05 (*), *p* < 0.001 (***)).

*n* = 28	BDI	BRI	MI	GEC
BRI	0.39 (0.047 *)			
MI	0.14 (0.503)	0.45 (0.021 *)		
GEC	0.30 (0.133)	0.81 (<0.001 ***)	0.86 (<0.001 ***)	
MinHR (min^−1^)	0.12 (0.546)	−0.11 (0.607)	0.15 (0.461)	0.02 (0.914)
AvHR (min^−1^)	−0.06 (0.764)	−0.01 (0.975)	0.18 (0.373)	0.09 (0.663)
HRV-Sleep (ms)	−0.21 (0.300)	−0.13 (0.519)	−0.44 (0.026 *)	−0.36 (0.071)
HRV-Awake (ms)	−0.23 (0.262)	−0.12 (0.550)	−0.40 (0.041 *)	−0.33 (0.105)
Steps	0.18 (0.371)	−0.05 (0.813)	−0.13 (0.511)	−0.07 (0.729)
Sleep (min)	−0.19 (0.362)	0.01 (0.976)	−0.27 (0.179)	−0.14 (0.489)

**Table 6:** Spearman´s partial rank correlation coefficients and statistical significances for BRIEF-A indices and Firstbeat^®^ results with gender and BBI-15 and BDI scores controlled for (*p* < 0.05 (*), *p* < 0.001 (***)).

*n* = 28	BRI	MI	GEC
MI	0.43 (0.030 *)		
GEC	0.79 (<0.001 ***)	0.87 (<0.001 ***)	
MinHR (min^−1^)	−0.17 (0.419)	0.14 (0.515)	−0.02 (0.938)
AvHR (min^−1^)	0.02 (0.927)	0.19 (0.356)	0.11 (0.587)
HRV-Sleep (ms)	−0.05 (0.794)	−0.42 (0.037 *)	−0.32 (0.122)
HRV-Awake (ms)	−0.04 (0.860)	−0.39 (0.057)	−0.28 (0.182)
Steps	−0.13 (0.525)	−0.16 (0.432)	0.14 (0.519)
Sleep (min)	0.09 (0.676)	−0.25 (0.222)	−0.09 (0.664)

## Data Availability

The data presented in this study are not available because of subjects’ privacy policy.
